# Seroprevalence and trend of human immunodeficiency virus among blood donors in Ethiopia: a systematic review and meta-analysis

**DOI:** 10.1186/s12879-019-4012-5

**Published:** 2019-05-06

**Authors:** Henok Mulugeta, Getenet Dessie, Fasil Wagnew, Dube Jara, Cheru Tesema Leshargie, Ayenew Negesse

**Affiliations:** 1 Department of Nursing, College of Health Science, Debre Markos University, P.O. Box 269, Debre Markos, Ethiopia; 2 Department of Nursing, School of health science, College of Medicine and Health Science, Bahir Dar University, P.O. Box 79, Bahir Dar, Ethiopia; 3 Department of Public Health, College of Health Science, Debre Markos University, P.O. Box 269, Debre Markos, Ethiopia; 4 Department of Environmental Health, College of Health Science, Debre Markos University, P.O. Box 269, Debre Markos, Ethiopia; 5 Department of Human nutrition and food science, College of Health Science, Debre Markos University, P.O. Box 269, Debre Markos, Ethiopia

**Keywords:** Blood donor, Human immunodeficiency virus, Seroprevalence, Ethiopia

## Abstract

**Background:**

Blood transfusion is one of the routine therapeutic interventions in hospitals that can be lifesaving. However, this intervention is related to several transfusion-related infections. The human immunodeficiency virus (HIV) is one of the major public health problems associated with blood transfusion. The objective of this systematic review and meta-analysis is to estimate seroprevalence and trend of human immunodeficiency virus among blood donors in Ethiopia.

**Methods:**

Studies on the prevalence of human immunodeficiency virus among blood donors published until 2017 were accessed by conducting a detailed search on PubMed, Cochrane Library, Google Scholar, EMBASE and CINAHL databases using the keywords:-“Seroprevalence” AND “trend” AND “HIV” OR “human” AND “immunodeficiency” AND “virus” OR “human immunodeficiency virus” AND “blood donors” OR “blood donors” OR “Ethiopia”. The quality of each article was assessed using a modified version of the Newcastle-Ottawa Scale. Meta-analysis was carried out using a random-effects method. All statistical analyses were done using STATA version 11 software.

**Result:**

The estimated pooled seroprevalence of human immunodeficiency virus among blood donors in Ethiopia was 2.69% (95% CI (1.79–3.58%)). The overall seroprevalence of human immunodeficiency virus infection showed a significant decline trend from 2004 to 2016.

**Conclusion:**

The overall seroprevalence of human immunodeficiency virus among blood donors in Ethiopia was high. Routine screening of donor blood for transfusion-transmissible infections is essential for ensuring the safety of blood transfusion.

## Background

The burden of Human immunodeficiency virus (HIV) remains one of the most serious public health concerns challenging the world. Worldwide, approximately 37 million people are infected with HIV. In Ethiopia, about 1.2 million people are living with HIV/AIDS and adult HIV prevalence in 2016 was estimated to be 1.1% [1, 2]. HIV can be transmitted through direct contact with blood, unprotected sexual intercourse with an infected person, use of contaminated needles and syringes, transfusion of infected blood, and mother to child during delivery and others [3, 4].

Blood transfusion is a routine therapeutic practice which is vital in increasing serum hemoglobin concentration of a patient with anemia and other various medical and surgical conditions. Although blood transfusion can be potentially lifesaving, the risk of several transfusions-transmissible infections such as human immunodeficiency virus (HIV), hepatitis B virus (HBV), hepatitis C virus (HCV) and syphilis is high. Screening of donor blood for transfusion-transmissible infections including HIV is essential for transfusion safety [5–7].

Transfusion of infected blood is one of the foremost causes of morbidity and mortality worldwide, particularly in sub-Saharan Africa, where it is responsible for 5–10% of new HIV infections [8]. Today, there is an increased need to ensure the safety of all donor blood within 72 h before transfusion [9]. However, the magnitude of transfusion-associated HIV transmission in sub-Saharan African countries remains high due to reduced financial resources and poor HIV antibody screening programs [10–12].

Maintaining a safe supply of blood for transfusion and blood products for all health facilities is the mission of the National Blood Bank Service of Ethiopia. This is in line to a recommendation from the WHO, which advises that all blood donations should be screened for infections prior to use [13].

In Ethiopia, several strategies have been implemented to ensure the safety of transfusable blood. Some of the strategies are: improving the operations of the Blood Bank Service, improving access to tools necessary for screening blood and blood products, careful donor selection criteria, safe screening and administration of blood to the recipients, and increasing public awareness on transfusion transmissible infections [14–17]. Although the safety of blood transfusion is maintained in most areas of the country, consistent screening practice is lacking in certain places, increasing the risk of major transfusion-transmissible infections such as HIV [18, 19].

Monitoring the seroprevalence HIV among blood donors is important for estimating the safety of blood and it is fundamental for developing various safety strategies to minimize infectious diseases transmission [7, 20]. Studies have been conducted worldwide to determine the prevalence of HIV among blood donors. For instance, seroprevalence of HIV among blood donors is 0.004% in Iran [3],0.08% in China [21], 0.07% in Saudi [22], 0.12% in Nepal [23], 0.002% in Turkey [24], 3% in Eastern Sudan [25], 3.1% in South-west Nigeria [26], 2.45 in Kenya [27], and 0.18% in Eritrea [28].

The few studies conducted in Ethiopia show that HIV infection is highly prevalent among blood donors. However, these findings are mostly inconclusive and inconsistent due to small sample sizes. Due to this, an accurate depiction of the national seroprevalence of HIV in blood donors remains unknown [4, 29]. Therefore, the objective of this systematic review and meta-analysis is to estimate the pooled seroprevalence of HIV among blood donors in Ethiopia.

## Methods

### Study design and literature search

Studies on the prevalence of HIV among blood donors published up to 2017 were searched using the following major databases: - PubMed, Cochrane Library, Google Scholar, CINAHL, and EMBASE. We followed the Preferred Reporting Items for Systematic reviews and Meta-Analyses (PRISMA) guidelines throughout the search [30]. EndNote version X7 (Thomson Reuters, London) was used to download, organize, review and cite the related articles. To obtain these articles we used the following search terms: “Seroprevalence”, “trend”, “HIV”, “human”, “immunodeficiency”, “virus”, “human immunodeficiency virus”, “blood donors”, and “blood donors “,” Ethiopia”. Boolean operators were used to combine search terms during the search as follows: Seroprevalence AND (“HIV”[MeSH Terms] OR “HIV” OR “human” AND “immunodeficiency” AND “virus” OR “human immunodeficiency virus” AND “blood donors”[MeSH Terms] OR “blood” AND “donors” OR “blood donors” AND “Ethiopia”.

### Data selection and eligibility

We included studies that reported the seroprevalence of HIV among blood donors in Ethiopia. Both published and gray literature reported in English language and studies published in the form master’s thesis and dissertations were also included. We excluded studies with unspecified sample size, studies that fail to report seroprevalence and studies conducted on the general population.

### Data extraction and quality assessment

The eligibility of all relevant articles that were included for full-text review was assessed by two reviewers (HM, GD). Whenever it was necessary, a third reviewer (FW) was consulted. Subsequently, all the selected papers were extracted using pre-piloted data extraction form in Microsoft Excel. The following study characteristics were recorded: author, year of publication, study area, health facility in which the study was conducted, the region of the country the study was conducted, study design, sample size, and the number of seropositive HIV individuals. Finally, all extraction data were checked by two other reviewers (DJ and AN) for accuracy and consistency. Any disagreement and inconsistencies were resolved by discussion and consensus. In this meta-analysis, the methodological and other qualities of the included article were assessed by using a modified version of the Newcastle-Ottawa Scale for cross-sectional studies adopted from PA Modesti et al. [31].

### Statistical analysis

We used a random effects model to generate the overall pooled estimate that is recommended to adjust for variability in the presence of heterogeneity among studies [32, 33]. This heterogeneity across studies was checked with I^2^ test statistics. Currently, I^2^ test statistics is the preferable and more reliable test in order to measure the variability (Heterogeneity) across the studies. I^2^ ranges between 0 and 100%. An I^2^ less than25% indicates homogeneous whereas I^2^ greater than or equal to 75% indicate very high heterogeneity across studies [34]. Further, the presence of heterogeneity was assessed by subgroup analysis. Data manipulation and statistical analyses were performed using the Statistical Software Package (STATA) Version 11.0(StataCorp, College Station, TX, USA).

### Assessment of publication Bias

Visual assessment of publication bias was conducted using a funnel plot. Asymmetrical distribution of studies on the funnel plot suggested the presence of publication bias [35]. Publication bias was also examined with Egger’s tests. Additionally, we performed a sensitivity analysis to assess whether the pooled prevalence estimates were influenced by individual studies.

## Results

### Search results and selection

**Fig. 1:**
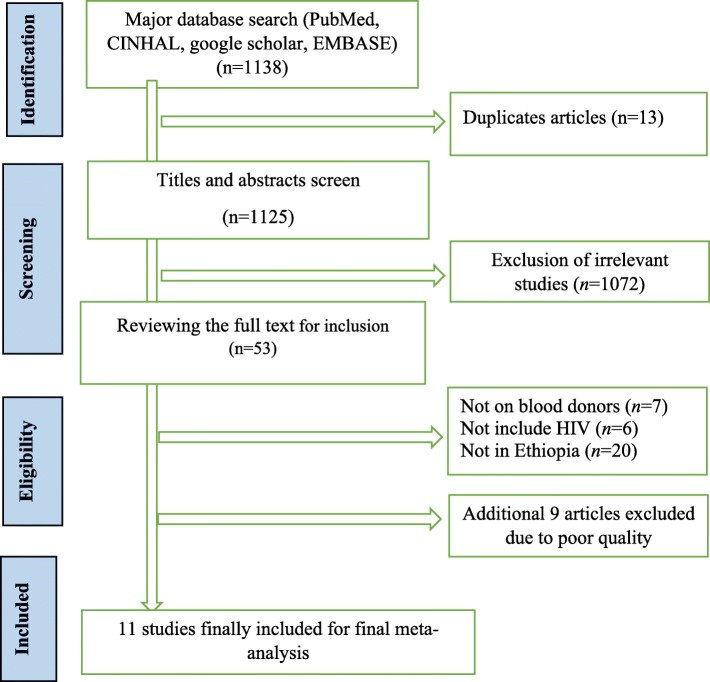
Flowchart of study selection for seroprevalence of HIV among blood donors

Initially, we identified 1138 studies in the electronic search process. Among these, 13 articles were duplicate and thus removed whereas 1072 were excluded after reviewing titles and abstracts. The full text of the remaining 53 studies was assessed for inclusion based on the predetermined inclusion criteria and 33 of these were excluded because they did not meet the eligibility criteria. Methodological quality assessment based on the Newcastle-Ottawa Scale resulted in the further exclusion of 9 articles. Finally, a total of 11 unique studies were included in the meta-analysis (Fig. 1).

### Characteristics of included studies

This systematic review and meta-analysis conducted up to 2017 included a total of 11 articles with 41, 845 participants in a representative sample from all over Ethiopia. The sample size of the studies ranged from 324 in a study conducted in Felege Hiwot Referral Hospital [36] to 9384 in Dessie Blood Bank [29]. Among 11 studies, four were conducted in the Amhara regional state [4, 14, 29, 36], two in Oromia regional state [37, 38], three in the South Nations Nationalities and Peoples (SNNP) region [18, 39, 40], and the remaining were carried out in the Somali region and Addis Ababa [41, 42]. All articles were cross-sectional studies. Characteristics of the included studies is summarized in Table 1.

**Table 1:** Characteristics of included studies for meta-analysis of HIV infection among blood donors

S.No	Author/s(reference)	Publication Year	Study Facility	Study area, Region	Study design	Sample size	Prevalence % (95% CI)
1	Azene Dessie, et al. [36]	2007	Felege Hiwot referral Hospital	Bahirdar, Amhara	Cross- sectional	324	11.7(8.2,15.2)
2	Bekele Sharew, et al. [29]	2017	Dessie Blood Bank	Dessie, Amhara	Cross- sectional	9384	5.1(4.65,5.55)
3	Belay Tessema, et al. [4]	2010	Gondar University Hospital	Gonder, Amhara	Cross- sectional	6361	3.8(3.33,4.27)
4	Belete Biadgo, et al. [14]	2017	Gondar District Blood Bank	Gondar, Amhara	Cross- sectional	6471	2.24(1.88,2.6)
5	Melese Gezahegn, et al. [37]	2012	Jimma University Hospital	Jimma, Oromia	Cross- sectional	3788	1(0.68,1.32)
6	Ramos, Jose M., et al. [38]	2016	Gambo Rural Hospital	Arsi, Oromia	Cross- sectional	2606	0.7(0.38,1.02)
7	Fisseha Bonja, et al. [40]	2017	Hawassa Blood Bank	Hawassa, SNNP	Cross- sectional	384	1.6(0.35,2.85)
8	Fithamlak Solomon, et al. [39]	2016	Wolaita Sodo University Hospital	Wolaita, SNNP	Cross- sectional	390	6.4(3.97,8.83)
9	Misganaw Birhaneselassie, et al. [18]	2016	Yirgalem Hospital Blood Bank	Yirgalem, SNNP	Cross- sectional	6337	1.6(1.29,1.91)
10	Yusuf M. and Alemayehu B. [42]	2016	Jijiga Bl ood Bank	Jijiga, Somali	Cross- sectional	4224	1.2(0.87,1.53)
11	Lulseged Assefa [41]	2017	National Blood Bank	Addis Abeba	Cross- sectional	1576	1.1(0.59,1.61)

### Prevalence of HIV infection among blood donors in Ethiopia (the result of meta-analysis)

**Fig. 2:**
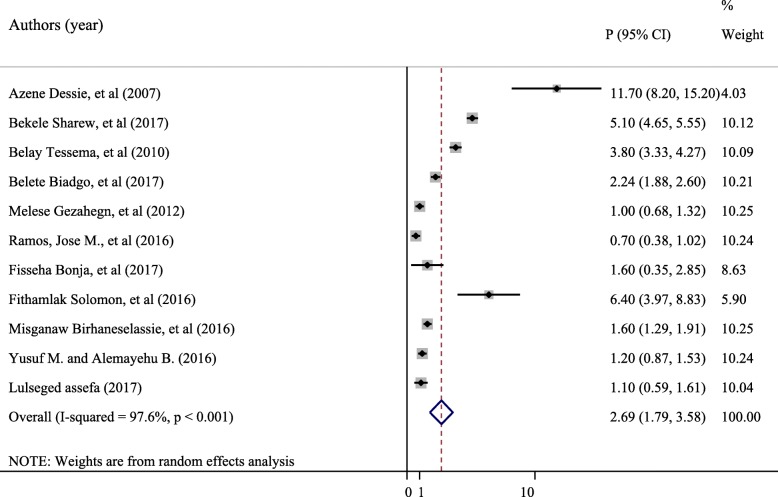
Forest plot showing the pooled seroprevalence of HIV among blood donors

The meta-analysis found significant heterogeneity across studies (I^2^ = 97.6%, *p* < 0.001), indicating that the fixed effect model may lead to an unreliable estimate. As a result, we used the random effects model to estimate the pooled seroprevalence of HIV among blood donors. The overall pooled prevalence of HIV reported by the 11 studies using the random effects model was 2.69% (95% CI: 1.79–3.58%). The pooled prevalence of HIV is presented in Fig. 2.

**Fig. 3:**
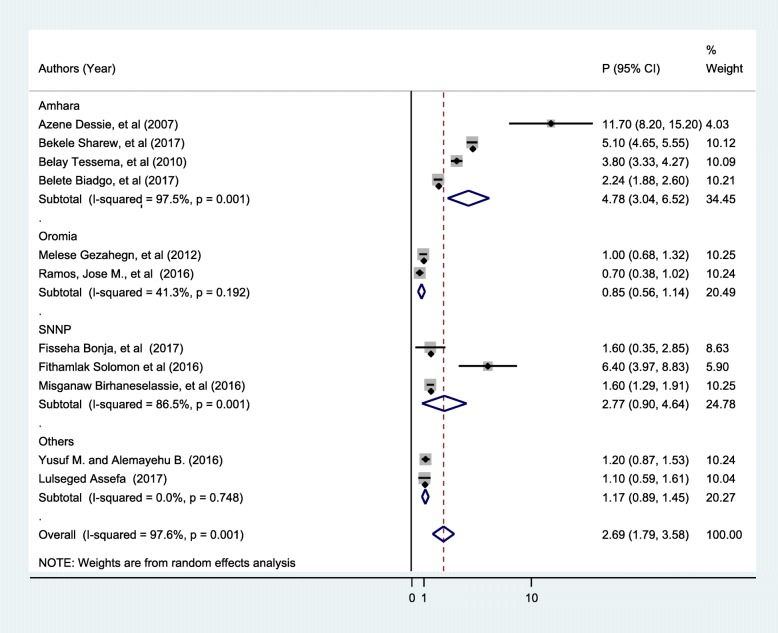
Subgroup analysis by regions on the seroprevalence of HIV among blood donors

In addition, we conducted sub-group analysis by region to minimize the potential random variations between studies by HIV seroprevalence among blood donors. The analysis found the highest estimated seroprevalence among blood donors among studies conducted in Amhara region (4.78, 95% CI: 3.04, 6.52%, I^2^ = 0.001). The subgroup analysis is presented in Fig. 3.

### Publication Bias assessment

**Fig. 4:**
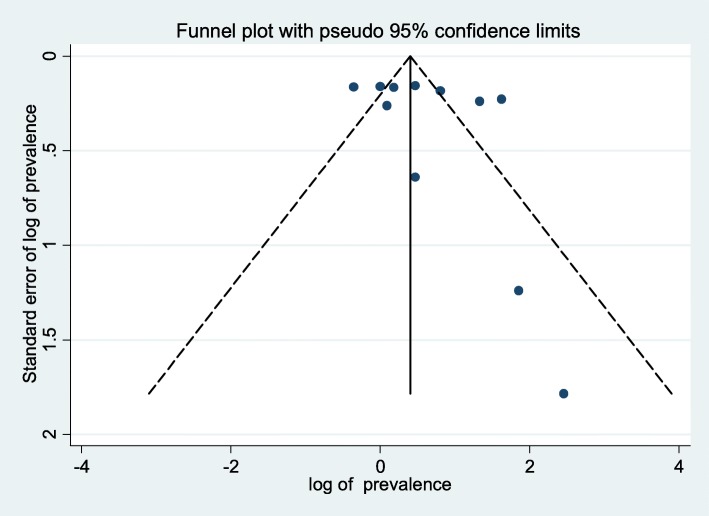
Funnel plot to test the publication bias in the 11 studies

**Fig. 5:**
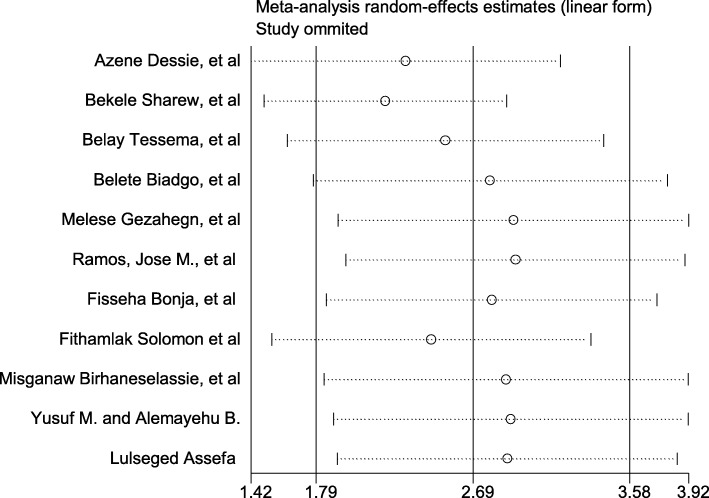
Result of sensitivity analysis of the 11 studies

Presence of publication bias was examined using a funnel plot and Egger’s test. Visual inspection of the funnel plot suggested asymmetry (Fig. 4). However, asymmetry of the funnel plot was not statistically significant as evidenced by Egger’s test (*P* = 0.31). Asymmetry in the funnel plots should not be always linked with publications bias [35]. High heterogeneity between the studies might be the reason for the asymmetry of the funnel plot in this systematic review and meta-analysis. Additionally, the result of sensitivity analyses using random effects model suggested that no single study unduly influenced the overall estimate (Fig. 5).

### Trend of HIV Seroprevalence among blood donors

**Fig. 6:**
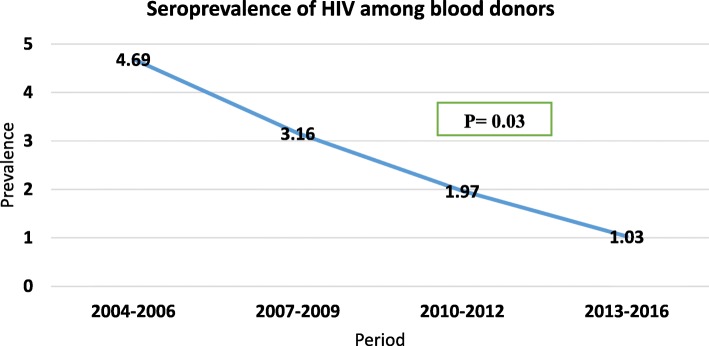
Trend of HIV prevalence among blood donors in Ethiopia from 2004 to 2016

Studies were divided into four groups according to the study period in order to observe the trend of HIV seroprevalence (Table 2). Our systematic review and meta-analysis showed that the seroprevalence of HIV among blood donors in Ethiopia has dramatically declined from 2004 to 2016. The seroprevalence of HIV was 4.69% between 2004 and 2006 and then decreased radically to 1.03% from 2013 to 2016. The trend of HIV prevalence among blood donors in Ethiopia is summarized in Fig. 6.

**Table 2:** Trend of HIV seroprevalence among blood donors in Ethiopia from 2004 to 2016

Study period	Prevalence of HIV % (95%CI)	Heterogeneity	
		I^2^	*P* value
2004–2006	4.69(3.31,6.08)	83.3	0.001
2007–2009	3.16(1.47,4.84)	95.2	0.001
2010–2012	1.97(1.32,2.62)	97.1	0.001
2013–2016	1.03(0.35,1.71)	89.2	0.001

### Distribution by gender and age group

Nine studies have tested the association between seroprevalence of HIV and sex of blood donors. The seroprevalence of HIV among male blood donors was 2.33(95%CI: 1.17, 3.48) and 2.65 (95%CI: 1.26–4.04) among female blood donors. However, there was no statistically significant difference in HIV seropositivity between male and female donors (*P* = 0.07). Additional three studies with similar age groups were investigated:-. HIV prevalence rate was 1.91% (95% CI: 0.17, 3.99) among blood donors aged 18–35), 2.86% (95% CI: 0.07–5.66) among blood donors aged 36–45, and 3.32% (95% CI: 2.16–4.48) among donors aged 45 or older.

## Discussion

Blood safety remains a major challenge in Africa, especially in Sub-Saharan countries [13]. Screening of blood has important public health benefits for the recipient and the community. Screening is also important for early prevention, diagnosis, and treatment of TTIs. The results of this study showed that the pooled prevalence of HIV infection among blood donors in studies spanning more than 12 years was 2.69% (95% CI (1.79–3.58%)). The prevalence of HIV infection found in this study is consistent with findings from similar ones conducted in different countries [7, 25–27]. Moreover, the seroprevalence of HIV among blood donors was unevenly distributed across the regions of the country, with the highest prevalence rate of HIV observed in Amhara regional state. This could be due to the fact that HIV incidence in the general population of Amhara region is high as compared to the other regions [43].

The WHO has estimated the prevalence of HIV in blood donors of low-income countries to be in the range of 0.56 to2.69% [13], which is comparable with our result. However, the overall estimate of HIV infection among blood donors in this study (2.69%) is higher than other similar studies [3, 21–24, 28, 44]. This higher prevalence rate might be attributable to low public awareness regarding HIV along with the higher incidence rate of HIV infection in the general population [45–47]. In contrast, studies conducted in some African countries such as in Equatorial Guinea, Nigeria, Ghana, and Tanzania showed the seroprevalence of HIV among blood donors was 7.83% [48], 4.2% [49], 4.06% [50], and 3.8% [51] respectively. These variations might be due to the difference in the eligibility criteria for blood donation, the type of donors and the effectiveness of the selection procedure.

Even though the incidence and the prevalence of HIV infection shows a rising pattern in the general population of the country in the last few years, the prevalence of HIV among blood donors in Ethiopia has declined from 2004 to 2016 (Fig. 6). Similar declining pattern has been reported among blood donors in United States [52], Iran [3] and Saudi Arabia [22]. The possible reason for this drastically declining trend in HIV seroprevalence throughout Ethiopia after 2004 might be the implementation of effective infection control measures such as the presence of highly qualified human resource, regular supply of reagents, and improved sensitivity of reagents used in blood screening tests in recent years [15]. However, global trends are not always decreasing as seen in China, where increasing HIV seroprevalence is reported [21], and in Ghana where seroprevalence of HIV infection among blood donors has remained steady [50].

Our findings also revealed that the seroprevalence of HIV was higher among female donors as compared to male donors. Other similar studies showed similar observation [21, 27, 49]. This could be due to the fact that females are more vulnerable in the general population than males for HIV infection due to different socioeconomic, cultural, and biological factors [53]. However, our findings are also contrary to other studies where the seroprevalence of HIV infection was higher among male donors than females [3, 23, 52, 54] or the same across the sexes [7].

This systematic review and meta-analysis also showed that the highest HIV seropositivity was detected on donors older than 45 years. This finding is in agreement with previous similar African studies reports [7, 48, 50]. This might be due to differences in risk behaviors among different age groups. Additional factors explaining the difference in age can be being away from home due to a job, increasing the risk of sexual contact. However, this finding was not in line with studies done in Nigeria [26] and Nepal [23] where a higher HIV seroprevalence rate was detected in younger age groups: 18–27 and 21–30 years respectively.

Although blood donors may not reflect the general population, this meta-analysis has provided valuable information regarding the seroprevalence of HIV among blood donors. However, there were some limitations that could be addressed in future systematic reviews and meta-analyses. The major limitation of this study is the lack of other published systematic reviews and meta-analyses on the seroprevalence of HIV among blood donors for comparison. Besides, because of the lack of similar variables in the selected articles, we were unable to analyze risk factors that can potentially explain the observed relationships.

The findings of this study have implication for clinical practice. Determining the seroprevalence of HIV among blood donors is critical to ensure the safety of blood transfusion. Therefore, ensuring the provision of safe blood transfusion requires effective legislative policies and strategies.

## Conclusion

This systematic review and meta-analysis show a high seroprevalence of HIV infection among Ethiopian blood donors. Hence, strict selections of blood donors with a standard clinical diagnosis and screening methods are highly recommended to ensure the safety of transfusable blood. However, this seroprevalence of HIV among blood donors is showing a declining trend across the years (2004 to 2016) in Ethiopia. HIV seropositivity is highly prevalent among females and on donors older than 45 years of age. Therefore, Ethiopian health authorities (e.g. Federal Ministry of Health) should strengthen the existing declining trend and design interventions to increase awareness among the aforementioned groups. Future large scale research is necessary to identify possible risk factors associated with HIV infection among blood donors.

## Abbreviations

AIDS: Acquired Immune Deficiency Syndrome
CI: Confidence Interval
HIV: Human Immune Deficiency Virus
PRISMA: Preferred Reporting Items for Systematic Reviews and Meta-Analyses
SNNP: Southern Nations, Nationalities, and Peoples
TTIs: Transfusion-Transmissible Infections
WHO: World Health Organization

## References

[1] CIA World Factbook (2018). Ethiopia HIV/AIDS - adult prevalence rate.

[2] World Health Organization (2018). Analytical summary - HIV/AIDS.

[3] Amini Kafi-abad S, Rezvan H, Abolghasemi H, Talebian A (2009). Prevalence and trends of human immunodeficiency virus, hepatitis B virus, and hepatitis C virus among blood donors in Iran, 2004 through 2007. Transfusion.

[4] Tessema B, Yismaw G, Kassu A, Amsalu A, Mulu A, Emmrich F, Sack U (2010). Seroprevalence of HIV, HBV, HCV and syphilis infections among blood donors at Gondar University teaching hospital, Northwest Ethiopia: declining trends over a period of five years. BMC Infect Dis.

[5] Moniri R, Mosayebii Z, Mossavi G (2004). Seroprevalence of cytomegalovirus, hepatitis B, hepatitis C and human immunodeficiency virus antibodies among volunteer blood donors. Iran J Public Health.

[6] Murphy M, Wallington T, Kelsey P, Boulton F, Bruce M, Cohen H, Duguid J, Knowles S, Poole G, Williamson L (2001). Guidelines for the clinical use of red cell transfusions. Br J Haematol.

[7] Nagalo MB, Sanou M, Bisseye C, Kaboré MI, Nebie YK, Kienou K, Kiba A, Dahourou H, Ouattara S, Zongo JD (2011). Seroprevalence of human immunodeficiency virus, hepatitis B and C viruses and syphilis among blood donors in Koudougou (Burkina Faso) in 2009. Blood Transfus.

[8] Allain JP, Owusu-Ofori S, Bates I (2004). Blood transfusion in sub-Saharan Africa. Transfus Altern Transfus Med.

[9] Cappellini M-D, Cohen A, Porter J, Taher A, Viprakasit V (2014). Guidelines for the management of transfusion dependent thalassaemia (TDT): Thalassaemia International Federation Nicosia, Cyprus.

[10] Field S. P, Allain J.-P. (2007). Transfusion in sub-Saharan Africa: does a Western model fit?. Journal of Clinical Pathology.

[11] Jayaraman S, Chalabi Z, Perel P, Guerriero C, Roberts I (2010). The risk of transfusion-transmitted infections in sub-Saharan Africa. Transfusion.

[12] Tagny CT, Mbanya D, Tapko JB, Lefrère JJ (2008). Blood safety in sub-Saharan Africa: a multi-factorial problem. Transfusion.

[13] World Health Organization: Blood safety and availability. 2017. Available on: https://www.who.int/news-room/fact-sheets/detail/blood-safety-and-availability.

[14] Biadgo B, Shiferaw E, Woldu B, Alene KA, Melku M (2017). Transfusion-transmissible viral infections among blood donors at the North Gondar district blood bank, Northwest Ethiopia: a three year retrospective study. PLoS One.

[15] WHO Guidelines Approved by the Guidelines Review Committee. In: Blood Donor Selection: GWHO Guidelines Approved by the Guidelines Review Committee. In: Blood Donor Selection: Guidelines on Assessing Donor Suitability for Blood Donation. edn. Geneva: World Health Organization

[16] Tapko J, Mainuka P, Diarra-Nama A (2006). Status of blood safety in the WHO African region. Report of the 2004 survey WHO-Afro Brazzaville, Congo.

[17] World Health Orginization (2017). Policy-makers in Ethiopia had a Forum to ensure an effective National Blood Transfusion System.

[18] Birhaneselassie M (2016). Prevalence of transfusion-transmissible infections in donors to an Ethiopian blood Bank between 2009 and 2013 and donation factors that would improve the safety of the blood supply in underdeveloped countries. Lab Med.

[19] Massenet D, Tesfaye G, Dandera B (1998). Blood transfusion in Ethiopia. Med Trop.

[20] Flichman DM, Blejer JL, Livellara BI, Re VE, Bartoli S, Bustos JA, Ansola CP, Hidalgo S, Cerda ME, Levin AE (2014). Prevalence and trends of markers of hepatitis B virus, hepatitis C virus and human immunodeficiency virus in argentine blood donors. BMC Infect Dis.

[21] Yang S, Jiao D, Liu C, Lv M, Li S, Chen Z, Deng Y, Zhao Y, Li J (2016). Seroprevalence of human immunodeficiency virus, hepatitis B and C viruses, and Treponema pallidum infections among blood donors at Shiyan, Central China. BMC Infect Dis.

[22] Elbjeirami WM, Arsheed NM, Al-Jedani HM, Elnagdy N, Hazem M (2015). Prevalence and Trends of HBV, HCV, and HIV Serological and NAT Markers and Profiles in Saudi Blood Donors. J Blood Disord Transfus.

[23] Shrestha AC, Ghimire P, Tiwari BR, Rajkarnikar M (2009). Transfusion-transmissible infections among blood donors in Kathmandu, Nepal. J Infect Dev Ctries.

[24] Uzun B, Güngör S, Demirci M (2013). Seroprevalence of transfusion transmissible infections among blood donors in western part of Turkey: a six-year study. Transfus Apher Sci.

[25] Abdallah TM, Ali AAA (2012). Sero-prevalence of transfusion-transmissible infectious diseases among blood donors in Kassala, eastern Sudan. J Med Med Sci.

[26] Buseri FI, Muhibi MA, Jeremiah ZA (2009). Sero-epidemiology of transfusion-transmissible infectious diseases among blood donors in Osogbo, south-West Nigeria. Blood Transfus.

[27] Wamamba D, Onyango D, Oyugi E, Kanyina E, Obonyo M, Githuku J, Ransom J (2017). Transfusion transmissible infections among walk-in blood donors at Kisumu regional blood transfusion Centre, Kisumu County, Kenya, 2015. Lab Med.

[28] Fessehaye N, Naik D, Fessehaye T. Transfusion transmitted infections - a retrospective analysis from the National Blood Transfusion Service in Eritrea. Pan Afr Med J. 2011;9:40. 10.4314/pamj.v9i1.71219 PMC3215562 22145069

[29] Sharew B, Mulu A, Teka B, Tesfaye T (2017). HIV-Sero-prevalence trend among blood donors in north East Ethiopia. Afr Health Sci.

[30] Moher D, Liberati A, Tetzlaff J, Altman DG, Group P (2009). Preferred reporting items for systematic reviews and meta-analyses: the PRISMA statement. PLoS Med.

[31] Modesti PA, Reboldi G, Cappuccio FP, Agyemang C, Remuzzi G, Rapi S, Perruolo E, Parati G (2016). Panethnic differences in blood pressure in Europe: a systematic review and meta-analysis. PLoS One.

[32] Barth RE, Huijgen Q, Taljaard J, Hoepelman AI (2010). Hepatitis B/C and HIV in sub-Saharan Africa: an association between highly prevalent infectious diseases. A systematic review and meta-analysis. Int J Infect Dis.

[33] White DL, Ratziu V, El-Serag HB (2008). Hepatitis C infection and risk of diabetes: a systematic review and meta-analysis. J Hepatol.

[34] Ried K (2006). Interpreting and understanding meta-analysis graphs: a practical guide.

[35] Tang J-L, Liu JL (2000). Misleading funnel plot for detection of bias in meta-analysis. J Clin Epidemiol.

[36] Dessie A, Abera B, Wale F (2007). Seroprevalence of major blood-borne infections among blood donars at Felege Hiwot referral hospital, Northwest Ethiopia. Ethiop J Health Dev.

[37] Gezahegn M, Woldemichae K, Godesso A (2012). HIV sero-prevalence trend among blood donors in Jimma University specialized hospital, Southwest Ethiopia. Ethiop J Health Sci.

[38] Ramos JM, Tissiano G, Fano H, Yohannes T, Gosa A, Reyes F, Górgolas M, Barreiro P (2016). Prevalence of positive HIV, HBV, HCV and treponemal tests in blood donors in a rural hospital in southern Ethiopia. J Clin Virol.

[39] Bisetegen FS, Bekele FB, Ageru TA, Wada FW (2016). Transfusion-transmissible infections among voluntary blood donors at Wolaita Sodo university teaching referral hospital**,** South Ethiopia. Can J Infect Dis Med Microbiol.

[40] Bonja F, Hussein M, Alemu J, Gemechu D, Birhaneselassie M (2017). The prevalence of transfusion transmitted infections: a focus on hepatitis B virus among blood donors at Hawassa blood bank center, southern Ethiopia. Int J Blood Transfus Immunohematol.

[41] Assefa L (2017). Sero-Prevalence Of Selected Transfusion-Transmitted Pathogen And Associated Risk Factors Among Blood Donors At The National Blood Bank Service In Addis Ababa.

[42] Mohammed Y, Bekele A (2016). Seroprevalence of transfusion transmitted infection among blood donors at Jijiga blood bank, eastern Ethiopia: retrospective 4 years study. BMC Res Notes.

[43] Ministry of Health (2017). National Comprehensive HIV Prevention, Care and Treatment Training for Health care Providers, training manual.

[44] Amiwero CE, Prescott RJ, George OA, Joy NI, Aisha M (2013). Seroprevalence of transfusion transmissible infections among blood donors attending the federal medical Centre, Bida. IJMBR.

[45] Haile Meskal F, Kefenie H, Selassie A, Kodakevich L (1994). A survey of harmful traditional practices in Ethiopia. Proceedings of the 11th International Conference of Ethiopian Studies: 1994.

[46] Kloos H, Mariam DH, Lindtjørn B. The AIDS epidemic in a low-income country: Ethiopia. Hum Ecol Rev. 2007:39–55.

[47] Shabbir I, Larson C (1995). Urban to rural routes of HIV infection spread in Ethiopia. J Trop Med Hyg.

[48] Xie D-D, Li J, Chen J-T, Eyi UM, Matesa RA, Obono MMO, Ehapo CS, Yang L-Y, Yang H, Yang H-T (2015). Seroprevalence of human immunodeficiency virus, hepatitis B virus, hepatitis C virus, and Treponema pallidum infections among blood donors on Bioko Island, Equatorial Guinea. PloS one.

[49] Mercy KA, Ijeoma I, Emmanuel KJ, Nwanyibuaku NG (2015). Prevalence of hepatitis B and human immunodeficiency virus co-infection among blood donors in Abia State University teaching hospital, aba, south east, Nigeria. Int J Curr Microbiol App Sci.

[50] Lokpo SY, Dakorah MP. The burden and trend of blood-Borne pathogens among asymptomatic adult population in Akwatia: a retrospective study at the St. Dominic Hospital, Ghana. 2017;2017:3452513. 10.1155/2017/3452513 PMC5664348 29181037

[51] Matee MI, Magesa PM, Lyamuya EF (2006). Seroprevalence of human immunodeficiency virus, hepatitis B and C viruses and syphilis infections among blood donors at the Muhimbili National Hospital in Dar Es Salaam, Tanzania. BMC Public Health.

[52] Zou S, Dorsey KA, Notari EP, Foster GA, Krysztof DE, Musavi F, Dodd RY, Stramer SL (2010). Prevalence, incidence, and residual risk of human immunodeficiency virus and hepatitis C virus infections among United States blood donors since the introduction of nucleic acid testing. Transfusion.

[53] Ramjee G, Daniels B (2013). Women and HIV in sub-Saharan Africa. AIDS Res Ther.

[54] Sube KL, Gore RP, Jaja S, Loro RL, Lino EO, Seriano OA, Wani SN, Alex LJ, Jack KR, Seriano OF (2014). Prevalence of HIV among blood donors at juba teaching hospital blood Bank, South Sudan. South Sudan Medical Journal.

